# A phylogeographic study of two acanthocephalan species from aquatic birds distributed in the Nearctic and neotropical region of Mexico and the USA

**DOI:** 10.1017/S0031182025100565

**Published:** 2025-09

**Authors:** Ana Lucia Sereno-Uribe, Marcelo Tonatiuh González-García, Alejandra López-Jiménez, Yeraldin Aldama-Prieto, Mirza Patricia Ortega-Olivares, Martín García-Varela

**Affiliations:** 1 Departamento de Zoología, Instituto de Biología, Universidad Nacional Autónoma de México, Ciudad de México, México; 2 Departamento de Biología Comparada, Facultad de Ciencias, Universidad Nacional Autónoma de México, Ciudad de México, México

**Keywords:** Acanthocephalans, Molecular markers, Phylogeography, *Polymorphus brevis*, *Pseudocorynosoma constrictum*

## Abstract

Acanthocephalans, which are in the family Polymorphidae, are a globally distributed group of endoparasites whose adults reside in the intestines of fish-eating birds, waterfowl and marine mammals. Adults of *Polymorphus brevis* and *Pseudocorynosoma constrictum* are endoparasites of fish-eating birds (Ardeids) and waterfowl (Anatidae), respectively, and are considered one of the most abundant and widely distributed species of polymorphids in freshwater systems from the Nearctic and Neotropical regions of Mexico and the USA. In the present study, sequences of cytochrome *c* oxidase subunit 1 (*cox1*) from mitochondrial DNA were generated from 67 specimens of *P. brevis* and 32 of *Ps. constrictum* from 12 localities on 6 biogeographic provinces in Mexico (the Trans-Mexican Volcanic Belt, Pacific Lowlands, Veracruzan, Californian, Sierra Madre Occidental, and Sonoran), plus the Temperate Prairies biogeographical province in the USA. The phylogeographic analyses indicated that the populations of both species lacked phylogeographic structure and exhibited high haplotype diversity, low nucleotide diversity and low Fst values among the biogeographic provinces; in combination with negative values in the neutrality test, these findings suggest that the populations of both species of acanthocephalan are undergoing expansion. The current evidence indicates that the biology of the definitive hosts, in combination with their migration patterns, could play a key role in shaping the distribution of haplotypes and the population genetic structure of the studied 2 acanthocephalan species.

## Graphical abstract

**Figure:**
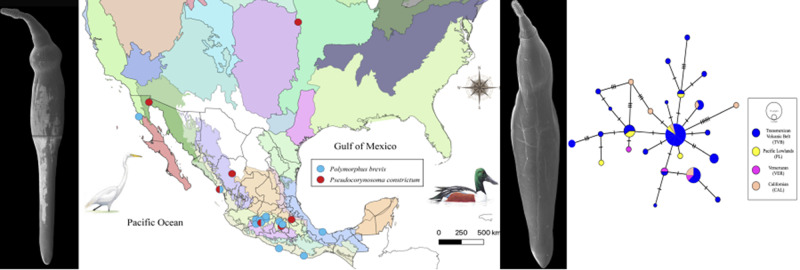

## Introduction

The phylogeography and distribution of any species are shaped by abiotic and biotic factors. In parasites that infect wild animals, the transmission mode, life cycle, dispersal and type of intermediate and definitive hosts play crucial roles in the genetic structure and diversity of species (Criscione and Blouin, 2004; Goulding and Cohen, 2014; van der Mescht et al., 2015; Perrot-Minnot et al., 2018; García-Varela et al., 2023). The recent application of molecular markers has provided insight into the taxonomy, systematics, delimitation species and phylogeographic structure of parasites, including acanthocephalans (Steinauer et al., 2007; Rosas-Valdez et al., 2012, 2020; Alcántar-Escalera et al., 2013; Goulding and Cohen, 2014; Perrot-Minnot et al., 2018; Pinacho-Pinacho et al., 2018; García-Varela et al., 2021, 2023; Sereno-Uribe et al., 2022). Members of the family Polymorphidae Meyer, 1931, are a globally distributed group of acanthocephalans whose adults reside in the intestines of fish-eating birds, waterfowl and marine mammals. The encysted cystacanth (larval form) resides in the body cavities of diverse crustaceans (amphipods, decapods and euphausiids) that serve as intermediate hosts to complete their life cycle. However, teleost fishes (paratenic hosts) play a principal role in transmission because they serve as ecological bridges facilitating infection to appropriate definitive hosts (Nickol et al., 1999, 2002; Aznar et al., 2006; Kennedy, 2006; García-Varela et al., 2013; Presswell et al., 2018, 2020). Currently, Polymorphidae contains 16 accepted genera based on morphological, ecological and molecular characteristics (Schmidt, 1973, 1975; Amin, 2013; García-Varela et al., 2013; Presswell et al., 2018, 2020; Ru et al., 2022; Rothman et al., 2025). Of the approximately 140 described species classified in Polymorphidae, 41 belong to the genus *Polymorphus* Luhë 1911, and 6 belong to *Pseudocorynosoma* Aznar, Pérez Ponce de León and Raga, 2006. Adult *Polymorphus* and *Pseudocorynosoma* are parasites of aquatic birds and amphipods distributed across the globe (Aznar et al., 2006; Amin, 2013; García-Varela et al., 2017). To date, 4 species of *Polymorphus* (*P. trochus* Van Cleave, 1945, *P. minutus* (Goeze, 1782), *P. obtusus* Van Cleave, 1918 and *P. brevis* Van Cleave, 1916) and 3 species of *Pseudocorynosoma* (*Ps. constrictum* Van Cleave, 1918; *Ps. anatarium* Van Cleave, 1945; and *Ps. tepehuanesi*; García-Varela et al., 2017) have been recognized in both biogeographical regions of Mexico (García-Prieto et al., 2010; Alcántar-Escalera et al., 2013; García-Varela et al., 2017).

The current records indicate that *P. brevis* and *Ps. constrictum* are sympatrically distributed in Mexico and the USA. Adults of *P. brevis* have been associated with at least 5 fish-eating bird species from the family Ardeidae, including black-crowned night heron (*Nycticorax nycticorax* L.), little blue heron (*Egretta caerulea* L.), snowy egret (*E. thula* Molina), great egret (*Ardea alba* L.) and American bittern (*Botaurus lentiginosus* Rackett). Adults of *Ps. constrictum* have been documented in at least 8 waterfowl species from the family Anatidae, including green-winged teal (*Anas crecca* Gmelin), blue-winged teal (*Spatula discors* L.), cinnamon teal (*A. cyanoptera* Vieillot), Mexican duck (*A. diazi* Ridgway), gadwall (*A. strepera* L.), northern shoveler (*A. clypeata* L.), lesser scaup (*Aythya affinis* Eyton) and redheads (*Ay. americana* Eyton) (García-Prieto et al., 2010; García-Varela et al., 2013a, 2017). These definitive hosts are highly abundant in various biogeographical regions of Mexico and may play important roles in the phylogeographic structure of the populations of both studied species. During several surveys of parasites infecting aquatic birds in the Neotropical and Nearctic regions of Mexico, specimens of *P. brevis* and *Ps. constrictum* were obtained from their definitive hosts and characterized via an integrative taxonomic approach. In the present study, we generated and examined sequences of cytochrome c oxidase subunit I (*cox1*) from the mitochondrial DNA of specimens belonging to *P. brevis* and *Ps. constrictum* with the objective of studying the phylogeographic structure of both species of parasites from 6 biogeographic provinces (Trans-Mexican Volcanic, Pacific Lowlands, Veracruzan, Californian, Sierra Madre Occidental and Sonoran) in Mexico plus the Temperate Prairies biogeographical province.

## Materials and methods

### Specimen collection

**Figure 1.**
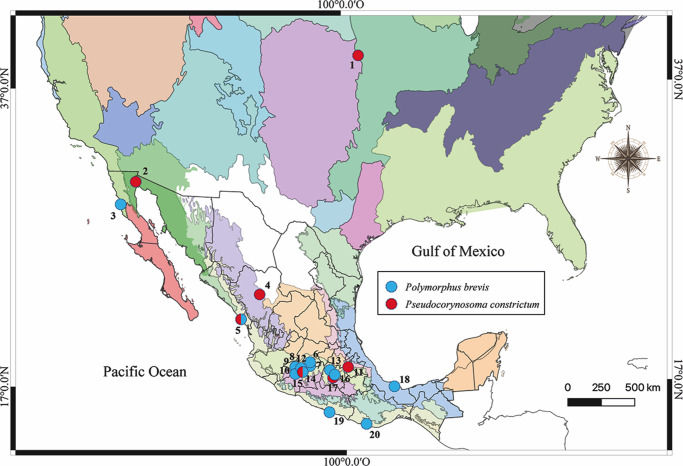
Map of Mexico showing the sampled sites for the birds. Localities with a circle of blue and red colour were positive for the infection with *Polymorphus brevis* and *Pseudocorynosoma constrictum* respectively; localities correspond to those in Table 1.

Aquatic birds were collected between October 2006 and December 2021 in 12 localities across 6 biogeographic provinces (Transmexican Volcanic Belt, Pacific Lowlands, Veracruzan, Californian, Sierra Madre Occidental, and Sonoran) from Mexico, plus Teperate Praires biogeographical province from USA (Table 1; Figure 1). Birds were dissected within the following 4 h, and their viscera were placed in separate Petri dishes containing a 0.75% saline solution and examined under a dissecting microscope. The acanthocephalans recovered were washed in 0.75% saline solution and placed distilled water at 4°C overnight and subsequently preserved in 70% ethanol. Birds were identified using the field guide of Howell and Webb (1995).

**Table 1.** Specimen information, collection sites, host, locality, geographical coordinates, GenBank accession number for specimens studied in this work. The sample number for each locality corresponds with the same number in the Figure 1. Sequences in bold were generated in the current study

Collection site	Species	Host	Locality	Coordinates North	West	GenBank Accession
1	*Pseudocorynosoma constrictum*	*Spatula discors*	Pawnee, Lake, Nebraska, USA	40° 50ʹ45.539ʹ’	96°52ʹ2.989ʹ’	**PV918601−607**
2	*Pseudocorynosoma constrictum*	*Anas clypeata*	San Luis Rio Colorado, Sonora	32°2ʹ12ʹ’	114°54ʹ07ʹ’	JX202535
3	*Polymorphus brevis*	*Botaurus lentiginosus*	San Quintin, Baja California	30° 24ʹ 57ʹ’	115°54ʹ72ʹ’	JX442194
						KC549451 − 452
						KC549479
						**PV918611 − 613**
4	*Pseudocorynosoma constrictum*	*Aythya affinis*	Santiaguillo, Durango	24° 49ʹ 45ʹ’	104° 53ʹ 16ʹ’	JX202530
						JX202527
						JX202538
5	*Pseudocorynosoma constrictum*	*Anas clypeata*	Caimanero, Sinaloa	23° 0ʹ 1.145ʹ’	106° 6ʹ 0.367ʹ’	JX202529
	*Polymorphus brevis*	*Nycticorax nycticorax*				**PV918614 − 616**
6	*Polymorphus brevis*	*Egretta caerulea*	Yuriria, Guanajuato	20° 13ʹ 16.495ʹ’	101°08ʹ11.14ʹ’	HM636467
						KC549446
						KC549496
						KC549470
7	*Polymorphus brevis*	*Nycticorax nycticorax*	Cuitzeo, Michoacan	19° 58ʹ 00.0ʹ’	101°07ʹ60ʹ’	DQ089717
						KC549466
						KC549447
						KC549450
						KC549469
						KC549471
						KC549474 − 475
						KC549477 − 478
						KC549482
						KC549487 − 488
						KC549490
8	*Polymorphus brevis*	*Zoogoneticus purepechus*	Las adjuntas, Michoacan	19° 54ʹ 39.3ʹ’	102° 12ʹ 20ʹ’	KC549448
9	*Polymorphus brevis*	*Zoogoneticus purhepechus*	Lago Camecuaro, Michoacan	19° 54ʹ 2.3ʹ’	102° 12ʹ 24.4ʹ’	KC549456
						KC549494 − 495
10	*Polymorphus brevis*	*Skiffia multipunctata*	Cupatziro, Michoacán	19° 52ʹ 51.3ʹ’	102° 12ʹ 34.7ʹ’	KC549455
						KC549449
						KC549497
11	*Pseudocorynosoma constrictum*	*Aythya americana*	Tecocomulco, Hidalgo	19°51ʹ26.05ʹ’	98° 23ʹ13.507ʹ’	JX202534
12	*Polymorphus brevis*	*Chirostoma humboldtianum*	Lago de Zacapu, Michoacán	19° 49ʹ 35ʹ’	101° 47ʹ 10ʹ’	KC549459
						KC549483
						KC549493
		*Alloophorus robustus*				KC549463
		*Notropis grandis*				KC549464
						KC549468
						KC549472
						KC549476
		*Zoogoneticus quitzeoensis*				KC549481
		*Skiffia lermae*				KC549492
13	*Polymorphus brevis*	*Girardinichthys multiradiatus*	Canal Santiago Tlacaque, Estado de México	19° 40ʹ 22ʹ’	99° 42ʹ 28ʹ’	KC549454
14	*Polymorphus brevis*	*Allophorus robustus*	Lago de Pátzcuaro, Michoacán	19° 33ʹ 5ʹ’	101° 40ʹ 6ʹ’	KC549453
						KC549480
						KC549491
		*Chirostoma estor*				KC549457
						KC549460
		*Goodea atripinnis*				KC549489
		*Zoogoneticus purhepechus*				KC549461
						KC549467
	*Pseudocorynosoma constrictum*	*Hyalella azteca*	Lago de Pátzcuaro, Michoacán	19° 33ʹ 5ʹ’	101° 40ʹ 6ʹ’	JX202531 − 533
						JX202536 − 537
						JX202543 − 544
15	*Polymorphus brevis*	*Allotoca catarinae*	Presa Caltzontzin, Michoacán	19° 25ʹ 14ʹ’	102° 12ʹ 22.0ʹ’	KC549458
						KC549465
						KC549473
						KC549484 − 486
16		*Girardinichthys multiradiatus*	Laguna Salazar, Estado de México	19° 21ʹ 05ʹ’	99° 21ʹ 55ʹ’	KC549462
17	*Pseudocorynosoma constrictum*	*Anas clypeata*	Almoloya, Estado de México	19° 09ʹ11.42ʹ’	99° 29ʹ 33.66ʹ’	EU267820
						JX202540
						**PV918608 − 610**
						JX202542
		*Anas cyanoptera*				JX202526
		*Anas strepera*				JX202528
		*Anas diazi*				JX202539
						KX688132
		*Anas crecca*				JX202541
						JX202545
18	*Polymorphus brevis*	*Egretta thula*	Catemaco, Veracruz	18° 25ʹ 00ʹ’	95° 07ʹ 00ʹ’	**PV918617 − 619**
19	*Polymorphus brevis*	*Egretta thula*	Tres Palos, Guerrero	16° 48ʹ 00ʹ’	99° 47ʹ 00ʹ’	EF467861
20	*Polymorphus brevis*	*Ardea alba*	Laguna de Manialtepec, Oaxaca	15°56ʹ 45ʹ’	97° 11ʹ 40ʹ’	**PV918620 − 621**

### Morphological analyses

Selected adult acanthocephalans were gently punctured in the trunk with a fine needle, stained with Mayer’s paracarmine, destained in 70% acid ethanol, dehydrated in a graded ethanol series, cleared in methyl salicylate and mounted in Canada balsam. Specimens were examined using a compound microscope Leica DM 1000 LED equipped with bright field (Leica, Wetzlar, Germany). The acanthocephalans were identified by conventional morphological criteria following Petrochenko (1958). In addition, descriptions of *P. brevis* and *Ps. constrictum* were consulted as needed (Van Cleave, 1945, 1945a). For scanning electron microscopy (SEM). Four adult specimens of each species were dehydrated with an ethanol series, critical point dried, sputter coated with gold, and examined with a Hitachi Stereoscan Model S-2469 N scanning electron microscope operating at 15 kV from the Instituto de Biología, Universidad Nacional Autónoma de México (UNAM). Adult specimens were deposited in the Colección Nacional de Helmintos (CNHE), Instituto de Biología, Universidad Nacional Autónoma de México, Mexico City, under the numbers 5720, 5778, 5881, 6270 and 6271.

### DNA isolation, amplification and sequencing

A total of 21 specimens, 11 identified morphologically as *P. brevis* and 10 as *Ps. constrictum* were placed individually in tubes and digested overnight at 56°C in a solution containing 10 mM Tris–HCl (pH 7.6), 20 mM NaCl, 100 mM Na_2_ EDTA (pH 8.0), 1% Sarkosyl, and 0.1 mg ml^−1^ proteinase K. Following digestion, DNA was extracted from the supernatant using the DNAzol reagent (Molecular Research Center, Cincinnati, Ohio) according to the manufacturer’s instructions. The cytochrome c oxidase subunit 1 (*cox 1*) of the mitochondrial DNA was amplified using the forward primer 5′-AGTTCTAATCATAA(R)GATAT(Y)GG-3′ and reverse primer 5′-TAAACTTCAGGGTGACCAAAAAATCA-3′ (Folmer et al., 1994). PCR reactions (25 μl) consisted of 10 μl of each primer, 2.5 μl of 10 × buffer, 2 mM MgCl_2_, and 1 U of Taq DNA polymerase (Platinum Taq, Invitrogen Corporation, São Paulo, Brazil). PCR cycling parameters for the molecular marker consisted of denaturation at 94°C for 1 min, 35 cycles of 94°C for 1 min, 40°C for 1 min and 72°C for 1 min, followed by a post-amplification incubation at 72°C for 10 min. Sequencing reactions were performed using ABI Big Dye (Applied Biosystems, Boston, Massachusetts) terminator sequencing chemistry, and reaction products were separated and detected using an ABI 3730 capillary DNA sequencer. Contigs were assembled and base-calling differences resolved using Codoncode Aligner version 12.0 (Codoncode Corporation, Dedham, Massachusetts) and submitted to GenBank dataset (Table 1).

### Alignments, population genetic structure and historical demographic

Newly obtained sequences in the current research of *P. brevis* were aligned with 56 other sequences of *P. brevis* (DQ089717, HM636467, JX442194, EF467861, KC549447 − 497) downloaded from GenBank (Table 1), forming a data set of 67 sequences with 615 characters. New sequences of *Ps. constrictum* were aligned with other sequences of *Ps. constrictum* (EU267820, KX688132, JX202526 − 545), downloaded from GenBank (Table 1), forming a data set of 32 sequences with 655 characters. Sequences of each dataset were aligned separately using the software Clustal W with default parameters implemented in MEGA version 7.0 (Kumar et al., 2016). To analyse the molecular information in the framework of population genetics, we grouped individuals of *P. brevis* and *Ps. constrictum* into populations considering the biogeographic provinces (Transmexican Volcanic Belt, Pacific Lowlands, Veracruzan, Californian, Sierra Madre Occidental). A single specimen of *Ps. constrictum* from Sonora (locality 2 in Table 1and Figure 1) was analysed together with seven specimens from the Temperate Praires biogeographical province in the USA (locality 1 in Table 1 and Figure 1). Intrapopulation variation was summarized using standard statistics: number of haplotypes (H), number of segregating sites (S), haplotype diversity (Hd), nucleotide diversity (Pi) and average number of nucleotide differences (K), were all calculated using the program DnaSP v. 5. 10 (Rozas et al., 2003). To examine haplotype frequency among the populations of *P. brevis* and *Ps. constrictum* a statistical network was constructed independently, using the program PopART with the median joining algorithm (Bandelt et al., 1999). The degree of genetic differentiation among the populations was estimated using the fixation indices Fst (Hudson et al., 1992), with the program Arlequin v.3.5 (Excoffier and Lischer, 2010). To investigate the population history and demography, Tajima’s D (Tajima, 1989) and Fu’s Fs (Fu, 1997) test were calculated using DnaSP v. 5. 10 (Rozas et al., 2003). The values were considered significant when the *P*-values were less than 0.05.

## Results

### Morphological identification

**Figure 2.**
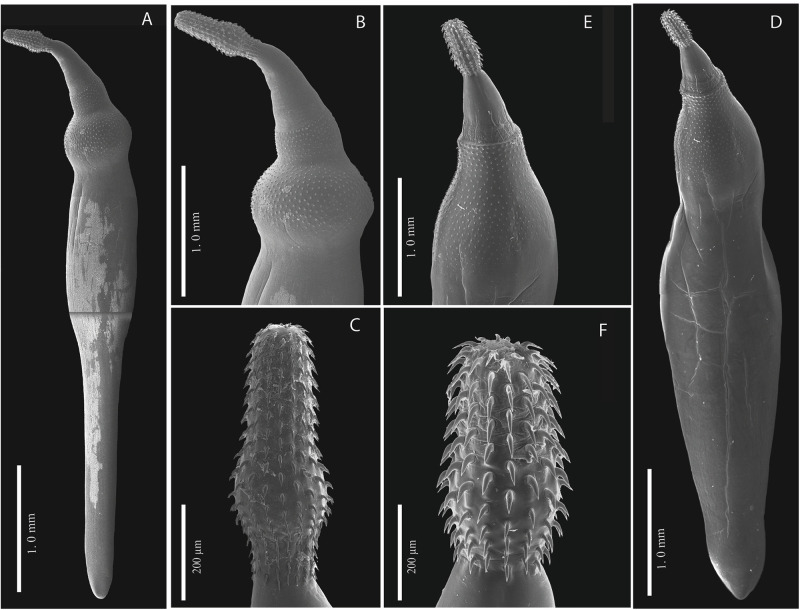
Scanning electron photomicrographs of *Polymorphus brevis* from *Botaurus lentiginosus* from San Quintin, Baja California, Mexico (locality 3 in Figure 1 and Table 1) and *Pseudocorynosoma constrictum* from *Anas clypeata* from Almoloya, Estado de México, Mexico (locality 17 in Figure 1 and Table 1). Adult male, whole worm (A, D); male anterior region (B, E); proboscis (C, F).

The body shape, somatic spine pattern in the trunk, proboscis hook number, proboscis shape, presence or absence of spines surrounding the genital pore and type of definitive host were compared to delineate the 2 species. For example, the acanthocephalans recovered from the intestines of 5 heron species showed similar morphological characteristics to those assigned to *P. brevis* by Van Cleave (1945) and Alcántar-Escalera et al. (2013), including an elongated cylindrical trunk with a single field of somatic spines on the anterior region of the trunk, a cylindrical proboscis with a swollen region, proboscis hooks arranged in 12–13 longitudinal rows of 17–19 hooks per row, a long neck, a double-walled proboscis receptacle, and four tubular cement glands in males (Figure 2A–C). The acanthocephalans recovered from the intestines of 8 waterfowl species presented morphological characteristics that matched those assigned to *Ps. constrictum* by García-Varela et al. (2013a, 2017). These included a cylindrical trunk with a single field of somatic spines on the anterior region of the trunk, with slight constriction separating the anterior and posterior regions of the trunk, a cylindrical proboscis recovering with 16 longitudinal rows of 10 hooks each, and a cone-shaped neck and genital spines surrounding the genital pore (Figure 2D–F).

### Population genetic structure and demographic analysis

**Figure 3.**
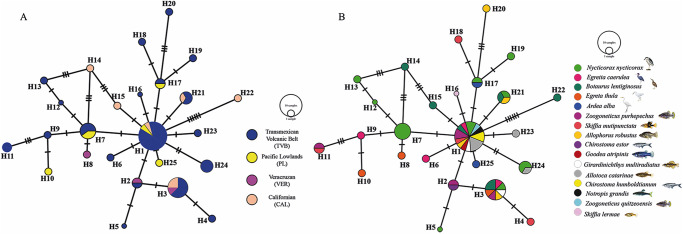
Haplotype network of samples of *Polymorphus brevis*, built with the gene cytochrome c oxidase subunit 1 (*cox1*) from mitochondrial DNA (A); host haplotype network (B). Each circle represents a haplotype, with size proportional to the haplotype’s frequency in the populations. Mutational steps are symbolized by dashes. biogeographic provinces, Trans-Mexican Volcanic Belt (TVM); Pacific Lowlands (PLN); Veracruzan (VER); Californian (CAL).

The mitochondrial marker was successfully amplified for 11 *P. brevis* individuals and 10 *Ps. constrictum* individuals. The complete alignment of the *cox1* dataset contained 67 *P. brevis* individuals with a total length of 615 bp, whereas the *cox1* dataset of *Ps. constrictum* contained 32 individuals with a total length of 655 bp. No insertions or deletions were detected in any of the sequences, and when the sequences were translated into proteins, no stop codons were found. The haplotype network built for *P. brevis* did not show a phylogeographic structure for the 26 mtDNA haplotypes detected. The most frequent haplotype (H = 1) was shared with the Trans-Mexican Volcanic Belt, Pacific Lowlands and Californian biogeographical provinces (Figure 3A). The identified haplotypes were separated for a few substitutions from 1, 2, 3 and 7 (see Figure 3A). The haplotype diversity was high (Hd = 0.885), and the nucleotide diversity was low (pi = 0.00404) among the populations from the 4 biogeographic provinces sampled (Trans-Mexican Volcanic Belt, Pacific Lowlands, Veracruzan, and Californian). Neutrality tests (Tajima’s D, − 1. 977 and Fu’s FS, − 19.625) were negative for all regions (see Table 2), which indicates an excess of rare alleles greater than what would be expected under neutrality, suggesting a recent population expansion of *P. brevis*. The Fst values were estimated to assess genetic differentiation among the populations from the 4 biogeographic provinces analysed. Despite the large geographic distances, the Fst values were low, ranging from 0.01 to 0.08 (average 0.00963) (Table 3), indicating that the populations were poorly genetically differentiated from one another.

**Table 2.** Molecular diversity indices and neutrality tests calculated for *cox1* data sets among the populations of *Polymorphus brevis* used in this study (n = number of sequences, H = number of haplotypes, S = number of segregating sites, hd = haplotype diversity, Pi = nucleotide diversity and K = average number of nucleotide differences). TVB = Transmexican Volcanic Belt; PL = Pacific Lowlands; VER = Veracruzan; CAL = Californian

Biogeographic provinces	n	S	H	Hd ± SD	Pi ± SD	K	Tajima’s D	Fu’s Fs
TVB	51	22	20	0.861 ± 0.038	0.00366 ± 0.00046	2.250	−1.735	−13.221
PL	6	5	5	0.933 ± 0.122	0.00314 ± 0.00078	1.933	−0.655	−2.186
VER	3	4	3	1 ± 0.272	0.00434 ± 0.00162	2.666	–	−0.341
CAL	7	14	6	0.952 ± 0.096	0.00743 ± 0.00182	4.571	−1.100	−1.311
All	67	31	26	0.885 ± 0.028	0.00404 ± 0.00045	2.487	−1.977	−19.625

**Table 3.** Pairwise fst values estimated for *cox1*. Significance level = 0.05. TVB = Transmexican Volcanic Belt; PL = Pacific Lowlands; VER= Veracruzan; CAL= Californian

	TVB	PL	VER	CAL
TVB	–			
PL	0.059	–		
VER	−0.047	0.080	–	
CAL	−0.019	0.057	−0.055	–

The host haplotype network revealed that some adult specimens of *P. brevis* recovered from 4 definitive hosts, namely, the black-crowned night heron (*N. nycticorax*), little blue heron (*E. caerulea*), snowy egret (*E. thula*) and American bittern (*B. lentiginosus*), share the same haplotype (H = 1). In addition, 17 haplotypes from adult specimens were scattered throughout the network (Figure 3B). The paratenic hosts harboured 9 haplotypes that were found in fishes belonging to the Atherinidae, Cyprinidae and Goodeidae families, suggesting that these hosts can harbour and transmit diverse haplotypes to their definitive hosts (Figure 3B).

**Figure 4.**
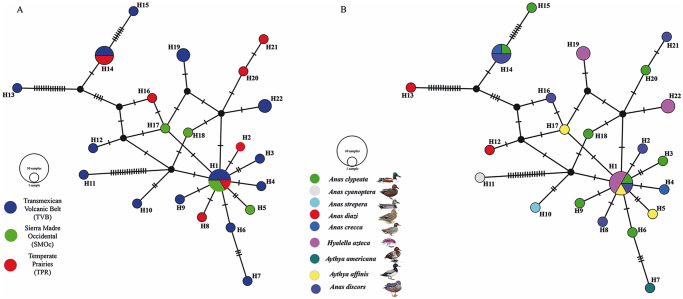
Haplotype network of samples of *Pseudocorynosoma constrictum*, built with the gene cytochrome c oxidase subunit 1 (*cox1*) from mitochondrial DNA (A); host haplotype network (B). Each circle represents a haplotype, with size proportional to the haplotype’s frequency in the populations. Mutational steps are symbolized by dashes. Biogeographic provinces, Trans-Mexican Volcanic Belt (TVM); Sierra Madre Occidental (smoc); Temperate Prairies (TPR).

The haplotype network built for the species *Ps. constrictum* did not show a phylogeographic structure among the 22 mtDNA haplotypes detected. The most frequent haplotype (H = 1) was shared with the Trans-Mexican Volcanic Belt, Sierra Madre Occidental and Temperate Prairies biogeographical provinces (Figure 4A). The haplotypes were separated for a few substitutions. However, the H11, H13 and H15 haplotypes presented seven to 15 substitutions (see Figure 4A). The haplotype diversity was high (Hd = 0.954), and the nucleotide diversity was low (pi = 0.01035) among the populations from the 3 biogeographic provinces sampled (the Trans-Mexican Volcanic Belt, Sierra Madre Occidental and Temperate Prairies). Neutrality tests (Tajima’s D, − 1.866 and Fu’s FS, − 8.844) were negative (see Table 4), suggesting an excess of rare alleles compared with what would be expected under neutrality, which is consistent with a recent population expansion of *Ps. constrictum*. The Fst values were estimated to assess genetic differentiation among the populations from the 3 biogeographic provinces analysed. Despite the large geographic distances, the Fst values were low, ranging from 0.03 to 0.08 (average 0.02804), which indicates that the populations were poorly genetically differentiated from one another (Table 5).

**Table 4.** Molecular diversity indices and neutrality tests calculated for *cox1* data sets among the populations of *Pseudocorynosoma* *constrictum* used in this study (n = number of sequences, H = number of haplotypes, S = number of segregating sites, hd = haplotype diversity, Pi = nucleotide diversity and K = average number of nucleotide differences). TVB = Transmexican Volcanic Belt; SMOc = Sierra Madre Occidental; TPR = Temperate Praires

Biogeographic provinces	n	S	H	Hd ± SD	Pi ± SD	K	Tajima’s D	Fu’s Fs
TVB	20	50	15	0.968 ± 0.025	0.01288 ± 0.00296	8.4368	−1.66373	−3.554
SMOc	4	3	3	0.833 ± 0.222	0.00229 ± 0.00084	1.50	−0.75445	−0.288
TPR	8	13	7	0.964 ± 0.077	0.00802 ± 0.00164	5.25	0.23858	−1.794
All	32	54	22	0.954 ± 0.024	0.01035 ± 0.00208	6.7762	−1.86640	−8.844

**Table 5.** Pairwise fst values estimated for *cox1*. Significance level = 0.05. TVB = Transmexican Volcanic Belt; SMOc = Sierra Madre Occidental; TPR = Temperate Praires

	TVB	SMOc	TPR
TVB	–		
SMOc	0.0669	–	
TPR	−0.0349	0.0847	–

The host haplotype network shows that the 8 waterfowl species sampled, namely, the green-winged teal (*A. crecca*), blue-winged teal (*S. discors*), cinnamon teal (*A. cyanoptera*), Mexican duck (*A. diazi*), gadwall (*A. strepera*), northern shoveler (*A. clypeata*), lesser acaup (*Ay. affinis*) and redheads (*Ay. americana*) are able to harbour multiple haplotypes of *Ps. constrictum*. In addition, the most frequent haplotype (H = 1) was shared with three definitive host species (Figure 4B). The intermediate host, the amphipod (*Hyalella azteca* Saussure), also harbours multiple haplotypes (H1, H19, H22), which are dispersed throughout the network (Figure 4B).

## Discussion

To the best of our knowledge, *P. brevis* and *Ps. constrictum* are 2 generalist species that use aquatic birds from the families Ardeidae (herons) and Anatidae (waterfowl) as definitive hosts and amphipods as intermediate hosts and are distributed sympatrically in Mexico and the USA (Amin, 1988; García-Prieto et al., 2010; Alcántar-Escalera et al., 2013; García-Varela et al., 2013a, 2017). The species *P. brevis* was described from the American bittern (*B. lentiginosus*) from Baltimore, Maryland (Van Cleave, 1916). Since then, *P. brevis* has been recorded as adults in 6 fish-eating birds from Louisiana and Florida in the USA (see Amin, 1988), as well as in 5 fish-eating birds from the Nearctic and Neotropical regions of Mexico (Alcántar-Escalera et al., 2013). In addition, cystacanths (larval form) of *P. brevis* have been found in 14 fish species from six families: Atherinidae, Cyprinidae, Goodeidae, Poeciliidae, Ictaluridae, Centrarchidae and Atherinopsidae (Amin, 1988; Alcántar-Escalera et al., 2013). Our specimens identified as *P. brevis* agree morphologically with those previously assigned by Van Cleave (1945) and Alcántar-Escalera et al. (2013), including an elongated cylindrical trunk with a single field of somatic spines on the anterior region of the trunk, a cylindrical proboscis with a swollen region, and proboscis hooks arranged on 12–13 longitudinal rows of 17–19 hooks per row (see Figure 2A–C).

The type species *Ps. constrictum* was described from the surf scoter bird (*Melanitta perspicillata* L.) from Yellowstone, Wyoming, USA (Van Cleave, 1945a), and since then, *Ps. constrictum* has been recorded in more than 20 waterfowl species and is considered one of the most abundant and widely distributed species of polymorphid in wetlands from the Nearctic region (Van Cleave, 1945a, Farias and Canaris, 1986; García-Varela et al., 2013a, 2017). Our specimens identified as *Ps. constrictum* agree morphologically with those previously described by Van Cleave, (1945a) and García-Varela et al. (2013a) because it has somatic spines covering the majority of the anterior part of the trunk, with slight constriction separating the anterior and posterior regions of the trunk, and it has a cylindrical proboscis with a slightly swollen region covered with 16 longitudinal rows of 10 hooks each (see Figure 2D–F).

The intraspecific genetic divergence estimated in the present study among the 67 isolates of *P. brevis* and the 32 isolates of *Ps. constrictum* analysed ranged from 0.00% to 1.8% (average 0.5%) and from 0.00% to 3.9% (average 1.0%), respectively. These values of intraspecific genetic divergence are similar to those previously reported for isolates of polymorphid species such as *Southwellina hispida* (Van Cleave, 1925) Witenberg, 1932, a parasite of herons, gulls, cormorants, pelicans and hawks, which presented divergence values ranging from 0.00% to 1.5%; *Hexaglandula corynosoma* (Travassos, 1915), a specialist species that has been recorded as an adult only in the intestine of the yellow-crowned night-heron (*Nyctanassa violacea* L.), ranging from 0.00% to 2.6% (García-Varela et al., 2023); and *Andracantha sigma* (Presswell, García-Varela et al., 2017), a parasite of seabirds and the Otago shag (*Leucocarbo chalconotus* Gray), spotted shag (*Phalacrocorax punctatus* Sparrman) and Otago little blue penguin, (*Eudyptula novaehollandiae* Forster) from New Zealand, which ranges from 0.00% to 0.32% (Presswell et al., 2018).

The haplotype network genealogy generated in this study was based on *cox1* sequences from *P. brevis* and *Ps. constrictum* and did not show a clear phylogeographic structure. As a result, the haplotypes could not be grouped according to their biogeographical provinces, despite the presence of geographical barriers such as mountains (Sierra Madre Occidental and Oriental), lowlands, drylands, the Balsas depression and the central Trans-Mexican Volcanic Belt (Barrier et al., 1998; Ferrari et al., 2012; Morrone et al., 2017). Interestingly, the lack of population genetic structure detected in the current study agrees with previous phylogeographic studies with other polymorphid species that have a broad distribution. For example, *Profilicollis altmani* (Perry, 1942), associated with multiple species of definitive hosts (gulls, ducks, sanderlings, and common tern); *Profilicollis novaezelandensis (*Brockerhoff and Smales, 2002), which has been found as adults in gulls (*Larus* spp.); and *Southwellina hispida*, associated with piscivorous birds throughout the world (see Brockerhoff and Smales, 2002; Goulding and Cohen, 2014; García-Varela et al., 2023).

Despite the considerable geographic distances, the Fst values estimated among the populations of *P. brevis* and *Ps. constrictum* were very low (Tables 3 and 5), indicating limited genetic differentiation. This pattern can be explained by the migration patterns of the definitive hosts herons and waterfowl, which can facilitate gene flow across regions. In addition, the estimated values of Fu´s Fs and Tajima´s D were negative, indicating that the populations of both acanthocephalan species may have undergone recent population expansion. *P. brevis* and *Ps. constrictum* are considered generalist species because they parasitize a broad spectrum of aquatic birds, mainly those of the families Ardeidae (herons) and Anatidae (waterfowl), which inhabit freshwater systems, such as ponds, lakes, rivers and wetlands in the Nearctic region, extending from central Mexico to the northern USA. It is well known that amphipods serve as intermediate hosts to *P. brevis* and *Ps. constrictum* (Amin, 1988; García-Varela et al., 2013a; Alcántar-Escalera et al., 2013). However, the main difference in the life cycles of the 2 species is the participation of the paratenic hosts: Freshwater fishes in the families Atherinidae, Cyprinidae, Goodeidae, Poeciliidae, Ictaluridae and Centrarchidae, which harbour the cystacanth (larval form), are key to the transmission of *P. brevis* to appropriate definitive hosts, whereas in the life cycle of *Ps. constrictum,* the infected amphipod (*H. aztecae*) exhibits notable behavioural and phenotypic shifts, including a bright orange spot in the haemocoel and altered photic behaviour, which increases predation by ducks (Bethel and Holmes, 1973; Benesh et al., 2005; Duclos et al., 2006).

Finally, the results of the present study allowed us to understand the genetic diversity and population genetic structure of two acanthocephalan species that parasitize a broad range of aquatic birds, mainly those of the families Ardeidae (herons) and Anatidae (waterfowl), which are distributed from central Mexico to the northern USA in the Nearctic region. Presumably, the biology and migration patterns of the definitive hosts, along with the involvement of paratenic hosts, may have played a key role in shaping the distribution of the haplotypes and the population genetic structure of the 2 acanthocephalan species studied.
